# Spatiotemporal Immune Dynamics in Experimental Retinal Ganglion Cell Injury Models

**DOI:** 10.1002/iid3.70284

**Published:** 2025-10-27

**Authors:** Chongqing Yang, Xiaoyu Wang, Xihang Ye, Ye Shen, Jianping Tong, Xuhong Zhang, Yudong Zhou

**Affiliations:** ^1^ Department of Ophthalmology of the First Affiliated Hospital and Zhejiang University School of Medicine Hangzhou China; ^2^ MOE Frontier Science Center for Brain Research and Brain‐Machine Integration, Nanhu Brain‐Computer Interface Institute Zhejiang University Hangzhou China; ^3^ School of Brain Science and Brain Medicine, Zhejiang University School of Medicine Hangzhou China; ^4^ Department of Psychiatry, the First Affiliated Hospital Zhejiang University School of Medicine, and the Zhejiang Key Laboratory of Precision Psychiatry Hangzhou China

**Keywords:** axonal injury, high intraocular pressure, immune cells, retinal ganglion cells, trauma

## Abstract

**Background:**

The damage and regeneration of retinal ganglion cells (RGCs) have been extensively studied. Among them, immune cells in different parts of the visual pathway play an important role in injury, regeneration and repair, but a comprehensive analysis of their spatial and temporal distribution is lacking.

**Purpose:**

This review emphasizes the unique characteristics of immune cells within the visual input pathway, focusing on their spatiotemporal dynamics in the retina, optic nerve head (ONH), and optic nerve during glaucoma and traumatic optic nerve injury.

**Methods:**

A comprehensive search was conducted across PubMed and Web of Science up to April 2025. Studies were included if they reported immune cells under glaucoma or optic nerve crush (ONC) animal models.

**Findings:**

Each region of the visual input pathway displays a distinct immune cell composition, including Müller cells, microglia, astrocytes, T cells, and oligodendrocytes, all of which work together to maintain homeostasis and respond to injury. Some immune cells are specific to certain regions, while others are shared across areas. Furthermore, even within a single glial cell type, there are different subtypes with unique developmental origins or marker profiles, reflecting a range of functions. In both glaucoma and traumatic optic nerve injury, retinal immune cells are rapidly activated, regardless of whether the initial impairment occurs in the soma or axon of RGCs, in the subacute or chronic course. The early stages of injury also see the presence of adaptive immune cells, such as T cells and neutrophils. Macrophages and microglia typically play complementary roles, while astrocytes show prolonged activation compared to microglia in the optic nerve, though this pattern does not hold in the retina following ONC.

**Conclusions:**

Understanding the spatiotemporal dynamics of these immune responses in glaucoma and traumatic optic nerve injury is crucial for developing targeted therapies that can reduce RGC loss, mitigate neurotoxicity, and promote functional recovery, ultimately preventing vision impairment. Targeting specific immune cell subsets may provide new strategies for delaying RGC damage.

## 1 Introduction

Retinal ganglion cells (RGCs) play a critical role in transmitting visual information to the brain, and damage to their cell bodies or axons can lead to irreversible vision loss [1]. The cell bodies of RGCs are situated in the retina, while their axons converge at the optic nerve head (ONH) and form the optic nerve [2]. These RGC axons project to various brain regions via the optic chiasm, thereby contributing to both visual and nonvisual complex signal processing [3]. Although the visual pathway is generally immune‐privileged (e.g., the existence of the blood‐ocular and blood‐retinal barriers [BRBs]), the distribution of immune cells along this pathway varies according to specific anatomical locations, cell types, and even subtypes, performing a diverse array of immune functions [4].

Immune responses generally manifest rapidly and can be sustained in the presence of stressors that inflict harm on RGCs, either directly or indirectly [5]. The distribution of RGC cell bodies and axons is extensive, and as such, different external stressors impact various parts of RGCs, resulting in distinct types of damage and progression [6]. Notably, elevated intraocular pressure (IOP) has been shown to impair axonal integrity at the ONH in the early stages of injury [7, 8]. In addition to alterations in axonal properties, the stiffness of RGC somas decreases, and their area shrinks under conditions of elevated IOP, either in a subacute or chronic course, although it is evident that trauma‐induced injury to RGCs primarily affects the axon [9]. Therefore, this review focuses on these two typical types of RGC injuries and examines the time‐dependent changes in immune responses.

The intricate interactions of immune cells in RGC axonal injury differ across ocular tissues, exhibiting significant heterogeneity in their composition and functions [10]. Various external stressors activate distinct types of immune cells; for instance, astrocytes and Müller cells are particularly responsive to mechanical stretch [11, 12], while microglia and infiltrating cells, such as macrophages and neutrophils, exhibit heightened activity in response to cellular debris [13]. The transition of cell types from pro‐inflammatory (e.g., type 1 astrocytes [A1] and type 1 macrophages/microglia [M1]) to anti‐inflammatory (e.g., type 2 astrocytes [A2] and type 2 macrophages/microglia [M2]) reflects distinct polarization modes in response to varied external stressors [14, 15]. Additionally, immune cells participate in various processes, including removal, repair, and protection of affected tissue, employing a range of cellular mechanisms such as secretion of inflammatory cytokines and phagocytosis of cellular debris [10, 16]. Another significant factor that merits consideration is the manner in which immune cells within a particular region respond over time to external stressors that exert an influence on another area, in conjunction with the aforementioned characteristics [17].

The objective of this review is to systematically compare the spatiotemporal dynamics of immune cells activated by RGC damage in different locations (soma and axon) within the contexts of glaucoma and traumatic optic nerve injury. The review emphasizes the distribution and functions of the immune cells across the retina, the ONH, and the optic nerve. Additionally, the review examines both acute and chronic immune responses exhibited by these cells in response to various external stressors, including fluctuations in IOP and trauma. The review further elucidates the complex interactions among immune cells, providing detailed insights into how these cells can simultaneously protect and compromise RGC function through well‐defined mechanisms.

## 2 Immune Cell Composition and Functional Differences in Retina, ONH, and Optic Nerve

**Figure 1:**
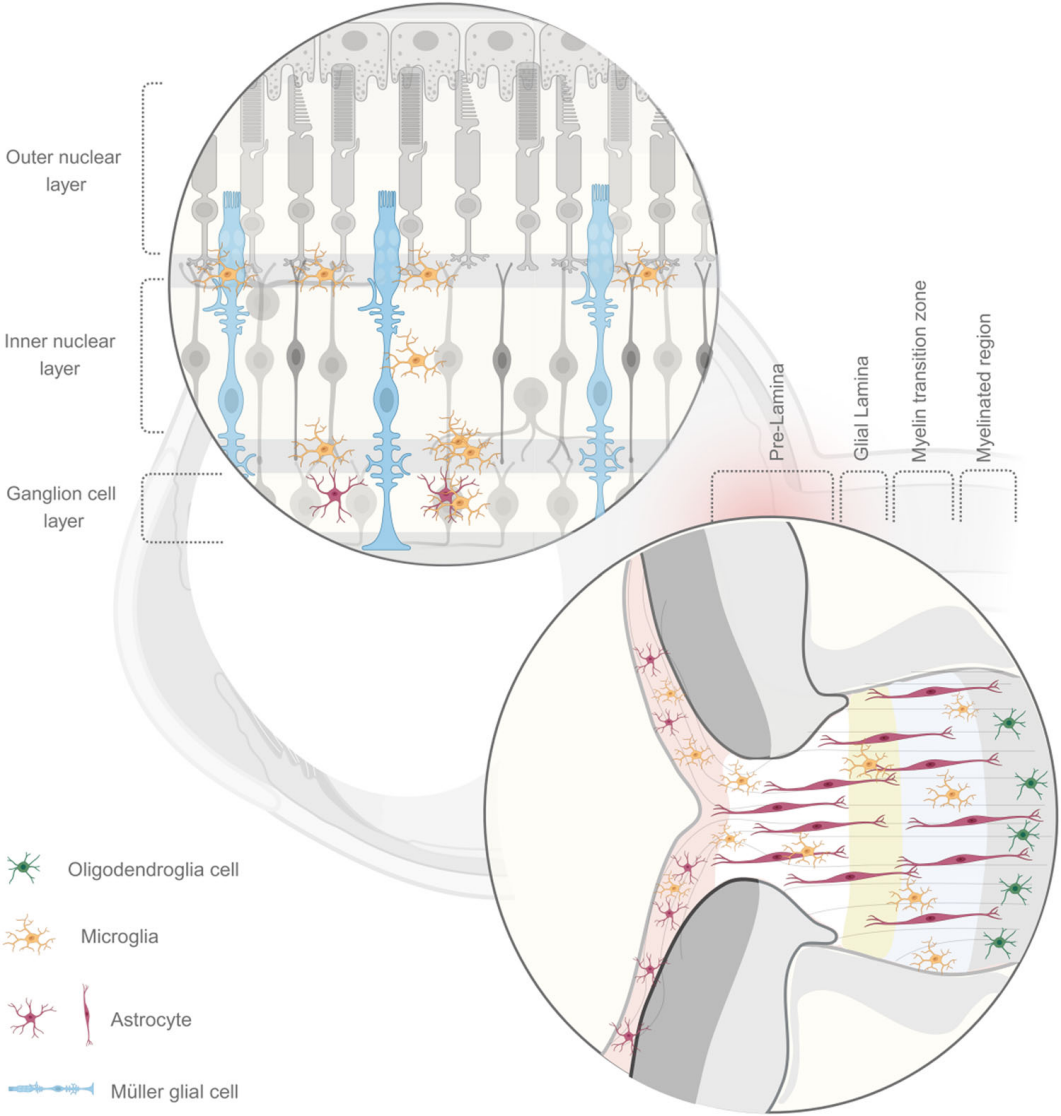
Distribution and morphological characteristics of major immune cells in the retina, optic nerve head, and optic nerve. The retina consists of distinct layers: the outer nuclear layer, outer plexiform layer, inner nuclear layer, inner plexiform layer, and ganglion cell layer. Similarly, the optic nerve head and optic nerve are organized into specific regions: the pre‐lamina, glial lamina, myelin transition zone, and myelinated region. The myelin transition zone encompasses the distal end of the unmyelinated optic nerve (UON) and is proximal to the myelinated region containing the myelinated optic nerve (MON). Müller cells span all retinal layers. Microglia are distributed within the retina, predominantly in the outer plexiform, inner plexiform, and inner nuclear layers, as well as throughout the optic nerve head and optic nerve, exhibiting similar morphology across these locations. Astrocytes reside in the retina, particularly the ganglion cell layer, and also populate the optic nerve head and optic nerve. Their morphology is stellate in both the retina and optic nerve head, but transitions to fibrillar in the optic nerve. Oligodendrocytes are found exclusively within the myelinated region.

**Figure 2:**
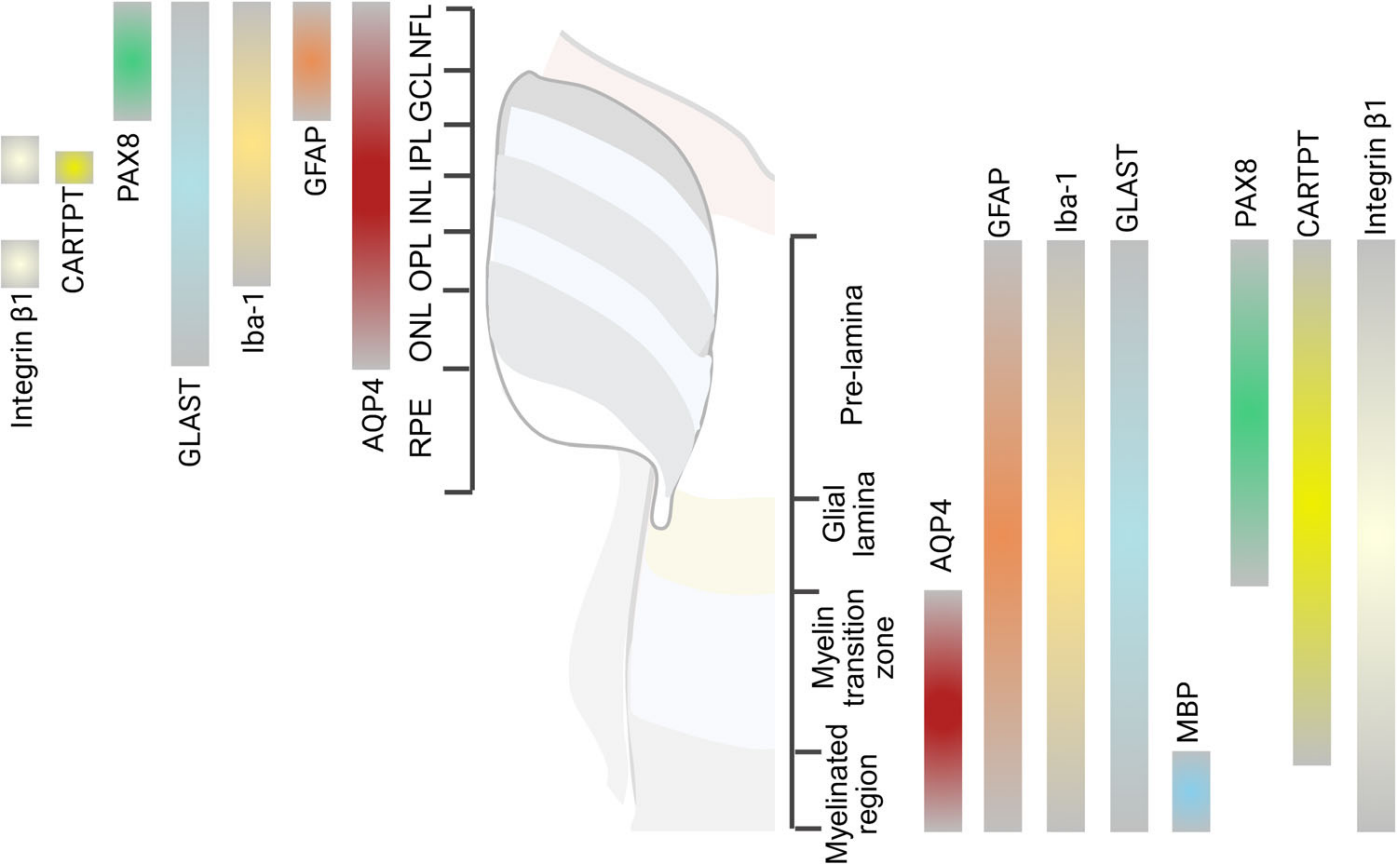
Distribution of key glial cell functional markers in the retina, ONH, and optic nerve. Most markers are co‐localized; however, some exhibit specific distributions. For instance, AQP4 is absent from the pre‐lamina and glial lamina. In contrast, PAX8 is predominantly present in these layers as well as in the GCL and NFL, suggesting a potential for functional complementation. MBP is found exclusively in the myelinated region, indicating its role as a specific myelin sheath marker. Integrin β1 and CARTPT demonstrate greater layer specificity within the retina compared to their expression in the optic nerve head and optic nerve. AQP4, aquaporin‐4; CARTPT, cocaine‐ and amphetamine‐regulated transcript prepropeptide; GCL, ganglion cell layer; GFAP, glial fibrillary acidic protein; GLAST, glutamate‐aspartate transporter; INL, inner nuclear layer; IPL, inner plexiform layer; MBP, myelin basic protein; NFL, nerve fiber layer; ONH, optic nerve head; ONL, outer nuclear layer; OPL, outer plexiform layer; PAX8, paired box 8; RPE, retinal pigment epithelium.

**Figure 3:**
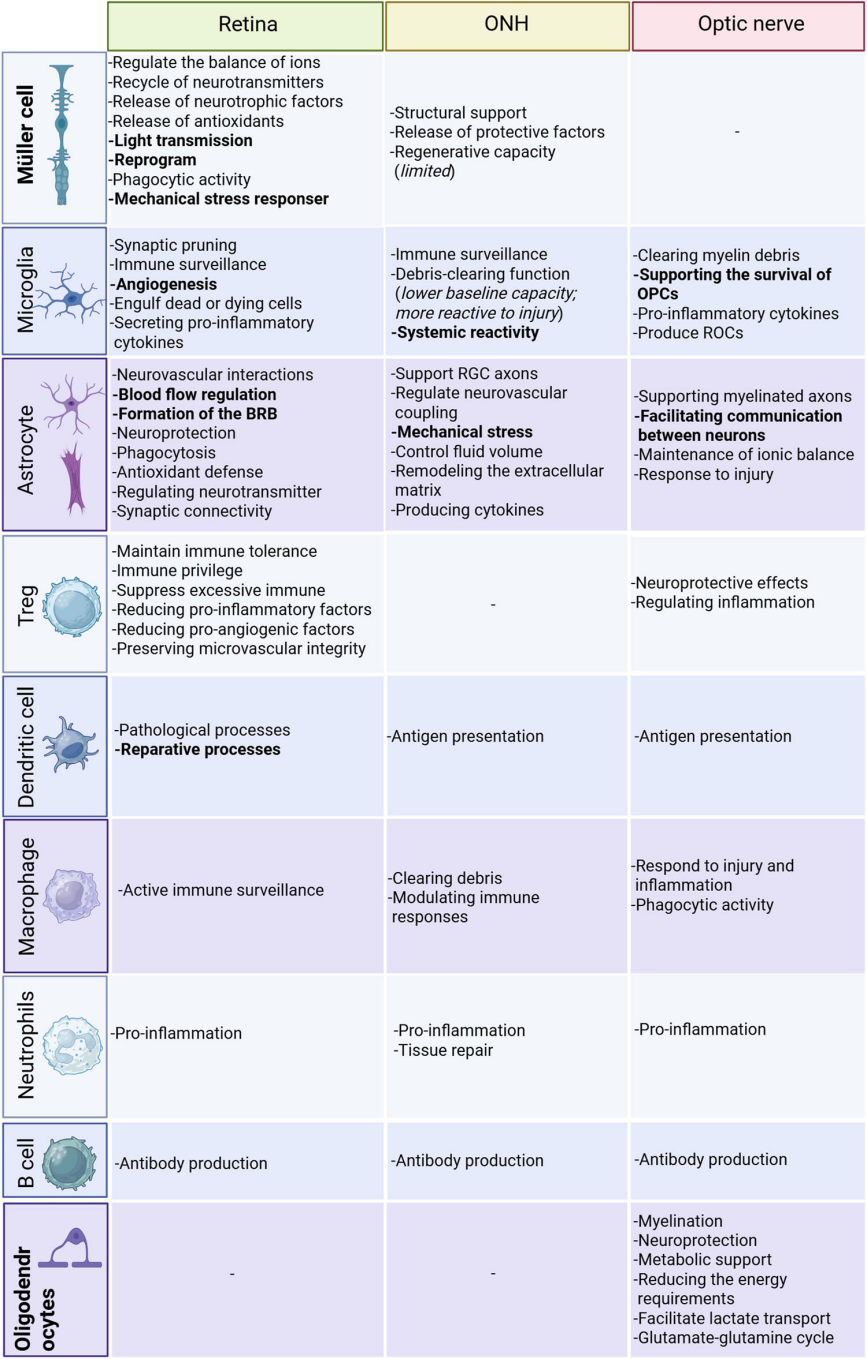
Key functions of immune and glial cells in the retina, optic nerve head, and optic nerve. Müller cells are unique to the retina, while oligodendrocytes are specific to the optic nerve, as indicated by the bold labels. Müller cells, microglia, astrocytes, and oligodendrocytes are all resident glial cells with diverse functions in their respective regions. For instance, Müller cells play distinct roles in light transmission, cell reprogramming, and mechanical responses within the retina. Microglia specifically regulate angiogenesis in the retina but exhibit systemic reactivity in the optic nerve head and support oligodendrocyte precursor cell (OPC) survival within the optic nerve. Astrocytes regulate blood flow and form the blood–brain barrier (BBB) in the retina, while in the optic nerve head, they handle mechanical stress, and in the optic nerve itself, they facilitate neuronal communication. These examples illustrate that identical cell types can perform different specific functions in distinct regions. Furthermore, the same function may be performed by different cell types in different areas. Finally, the degree to which cells perform a given function can also vary regionally (as indicated by the italics). In contrast, infiltrating immune cells tend to exhibit similar functions regardless of location.

The retina, the ONH, and the optic nerve collectively exhibit properties of immune privilege [18]. The hallmark features of these anatomical structures include the presence of blood‐ocular barriers and the absence of lymphatic drainage [19]. These structural features play a critical role in the preservation of vision and the prevention of inflammatory damage [19]. Therefore, specialized cells such as microglia are of significance in local immune regulation [20]. However, notable variations in the composition and functionality of immune and glial cells are observed within the retina, the ONH, and the optic nerve [5, 21, 22]. These disparities are attributable to the specific anatomical structure (Figure 1) and functions (Figures 2 and 3) of the aforementioned ocular tissues.

### 2.1 Immune Cell Composition and Function in the Retina

In the retina, under conditions of homeostatic balance, a complex population of immune and glial cells exists, including Müller cells, microglia, astrocytes, dendritic cells (DCs), and regulatory T cells (Tregs) [19, 23, 24, 25] (Figure 1).

Müller cells constitute the predominant glial cells in the retina, comprising over 90% of retinal glia in humans and mice [26]. These specialized radial glia extend throughout the entire thickness of the retina, interacting with all retinal cell types to maintain homeostasis [26] (Figure 1). These cells regulate the balance of ions, water, and bicarbonate; facilitate the recycling of neurotransmitters; and contribute to retinoid metabolism [27, 28]. Müller cells release neurotrophic factors and antioxidants, thereby protecting retinal neurons and supporting metabolic demands [29]. Their distinctive funnel‐like morphology, which facilitates light transmission to photoreceptors with minimal scatter, functions analogously to optical fibers [30]. Beyond their homeostatic role, Müller cells exhibit regenerative potential after injury by reprogramming into multipotent progenitors to aid in retinal repair [31]. They also display phagocytic activity, clearing apoptotic neurons and photoreceptor debris. This is often how they help out when there are not enough microglia [32]. Furthermore, Müller cells exhibit a remarkable capacity for adaptation in response to mechanical stress [33]. This capacity is evidenced by the activation of signaling pathways and the upregulation of stress‐responsive transcription factors [34, 35] (Figures 2 and 3).

Microglia, the resident innate immune cells of the retina, originate from yolk sac progenitors and constitute 5%–10% of retinal glia [36]. Under physiological conditions, microglia are predominantly localized to the ganglion cell layer (GCL), the inner plexiform layer (IPL), and the outer plexiform layer (OPL), with fewer present in the outer nuclear layer [37, 38] (Figure 1). In their resting state, they adopt a ramified morphology, performing functions such as synaptic pruning, immune surveillance, and angiogenesis [39]. Microglia transition to a reactive state in response to injury or disease, migrating to the damaged regions, engulfing cellular debris, and secreting pro‐inflammatory cytokines [40]. This early stage of microglia is commonly referred to as type 1 microglia (M1) [41]. Microglia of the M1 type are activated by damage‐associated molecular patterns (DAMPs) and cytokines such as interleukin‐1 beta (IL‐1β) and interferon‐gamma (IFN‐γ). They secrete various cytokines, including IL‐6 and IL‐12/IL‐23, as well as chemokines such as C‐C motif chemokine ligand 2 (CCL2) and C‐X‐C motif chemokine ligand 10 (CXCL10). During the middle and late stages of injury, type 2 microglia (M2), induced by IL‐4 and IL‐13, predominate. M2 microglia secrete anti‐inflammatory factors, such as IL‐10 and transforming growth factor‐beta (TGF‐β), and promote tissue debris clearance, angiogenesis, and nerve repair [41]. Microglia primarily engulf their own dead or dying cells, but they can also respond to other cellular debris when activated [42]. The capacity of microglia to clear debris is influenced by interactions with astrocytes, which may compete for the same substrates (Figure 3). More remarkably, microglial activation can now be directly observed in vivo. Scanning laser ophthalmoscopy combined with adaptive optics enables three‐dimensional localization and dynamic tracking of fluorescently labeled microglia [43]; additionally, swept‐source optical coherence tomography angiography paired with En‐face image processing has, for the first time, visualized and classified microglia and vitreous cells dynamically on the retinal surface in the living human eye [44].

Retinal astrocytes, classified as type Ib astrocytes, are found in high concentration in the vicinity of the ONH and are located in the GCL [45]. Derived from optic stalk precursors during development, they constitute approximately 0.1% of retinal cells, a figure significantly lower than that of Müller cells [46, 47]. Astrocytes exhibit a stellate morphology, with structural variations observed according to their location within the retina [48]. Recent studies have shown the potential for these cells to manifest intricate morphological patterns, which may serve as indicators of their neurovascular interactions and functions [48] (Figure 1). These cells have been observed in conjunction with pericytes and endothelial cells, contributing to blood flow regulation and the formation of the BRB [49]. They also exert neuroprotective effects and support RGCs by forming gap junction networks with Müller cells [50]. Furthermore, astrocytes engage in phagocytosis, thereby clearing microglial debris via complement‐ and autophagy‐dependent pathways [42]. The phagocytic rate of these cells exhibits a correlation with microglial turnover, particularly in circumstances of microglial activation or death. Moreover, astrocytes provide antioxidant defense, thus protecting neurons from oxidative damage [51]. Astrocytes also play a role in the maintenance of neural circuits and retinal homeostasis by regulating neurotransmitter uptake and synaptic connectivity [52] (Figures 2 and 3).

Tregs are a subset of CD4^+^ T cells that maintain immune tolerance and retinal immune privilege. These cells have the capacity to suppress excessive immune responses to self and environmental antigens, in part by reducing pro‐inflammatory and pro‐angiogenic factors [53]. Tregs have been shown to interact with microglia, thereby preserving retinal microvascular integrity and facilitating the repair of neovascularization and vascular leakage [54] (Figure 3).

DCs in the retina are primarily perivascular and localized to the GCL and the nerve fiber layer (NFL), often in proximity to large blood vessels [55]. Highly branched DCs are associated with smaller vessels, while few are found in the IPL or absent in the inner nuclear layer (INL) [56]. It is noteworthy that DCs are predominantly implicated in pathological or reparative processes as opposed to their role in routine retinal function [57] (Figure 3).

Macrophages are also observed in the retina, particularly on the inner limiting membrane [58]. In healthy eyes, the distribution of these cells exhibits a sparse center and denser periphery, with a decrease in density with age. These macrophages exhibit dynamic behaviors, such as rapid (several minutes) process extension and contraction, suggesting active immune surveillance [41]. The majority of CD45^+^CD11b^+^ cells in the retina are microglia, but small populations of macrophages (Fcgr1^+^C1qa^+^) expressing genes like membrane spanning 4 domains A7 and apolipoprotein E have also been identified [59]. The hallmark of these cells is the expression of specific markers, including CD68, which serves as an indication of phagocytosis [60, 61] (Figure 3).

In summary, retinal glial and immune cells exhibit distinct but overlapping roles: Müller cells primarily maintain homeostasis and metabolism, microglia regulate immune surveillance and phagocytosis, astrocytes support vascular health and neuroprotection, and Tregs uphold immune privilege. Collectively, these cells constitute a finely tuned network that sustains retinal structure and function.

### 2.2 Immune Cell Composition and Function in ONH

The ONH, otherwise known as the optic disc, serves as a critical junction where the axons of RGCs converge to form the optic nerve [62]. This anatomical structure is distinguished from the retina both structurally and functionally [62]. Its thickness and subareas vary across species [63]. The ONH is home to a variety of immune and glial cells, including astrocytes, microglia, DCs, macrophages, and neutrophils [64]. Collectively, these cells maintain the health of the ONH and respond to injury or stress. While some cell types exhibit functional overlap with their retinal counterparts, others manifest unique characteristics adapted to the specific environmental demands of the ONH (Figures 2 and 3).

Astrocytes in the ONH, classified as type Ia [45], differ from retinal astrocytes in morphology and molecular composition. These fibrous astrocytes are characterized by their flattened, polygonal shape with elongated processes that interweave among RGC axons, particularly in the vicinity of the lamina cribrosa [50] (Figure 1). Similar to retinal astrocytes, these ONH astrocytes support RGC axons, regulate neurovascular coupling [50], and respond to mechanical stress by altering their morphology and reactivity [65]. In doing so, they express mechanosensitive ion channels [66]. In response to injury, they regulate free water bidirectional flow and control fluid volume, remodeling the extracellular matrix (ECM), and producing specific cytokines that promote cell surface molecular expression [67] (Figure 3). However, in contrast to retinal astrocytes, ONH astrocytes do not express aquaporin‐4 (AQP4), a water channel protein. This absence is thought to restrict fluid transfer at the lamina cribrosa, thereby maintaining the pressure gradient necessary for optic nerve function [68]. This unique adaptation also underscores the specialized role of ONH astrocytes in fluid regulation, distinguishing the ocular glymphatic system from its brain counterpart [69] (Figure 2).

The distribution of microglia in the ONH is characterized by a notable degree of variability, with a tendency to accumulate in proximity to blood vessels and nerve bundles [70] (Figure 1). The precise population size of microglia in the ONH can exhibit significant variations based on several factors, including age, health status, and injury. Under typical conditions, microglia maintain a stable population with minimal turnover [70]. While they share core immune surveillance and debris‐clearing functions with retinal microglia, ONH microglia have a lower baseline phagocytic capacity [71] and are more reactive to injury. Interestingly, microglia in the contralateral eye may also respond to ONH injury, highlighting their systemic reactivity [72] (Figure 3).

Müller cells, which are primarily located in the peripapillary region and retinal nerve fiber layer (RNFL) near the ONH, are also found in the middle of the ONH, labeled by SRY‐box transcription factor 9 [73], differ in their behavior compared to retinal Müller cells [74] (Figures 1 and 2). While both contribute to structural support and the release of protective factors under stress, Müller cells in the ONH exhibit limited regenerative capacity and lack the ability to transdifferentiate into neurons [75]. This feature is more prominent in the retina and lower vertebrates. Approximately 20% of the RNFL in the vicinity of the ONH consists of non‐axonal components, including Müller cells, indicating their structural importance in this region [74] (Figure 3).

The ONH also contains a higher density of DCs, macrophages, and neutrophils, as well as plasma cells and B cells, in comparison to the retina [76]. DCs, which are primarily associated with antigen presentation, are concentrated in regions where the BRB is less developed [5, 77]. Macrophages infiltrate the ONH during inflammatory events, clearing debris and modulating immune responses [5, 78]. Neutrophils, while present in low numbers under normal conditions, become more prevalent during injuries or infections, contributing to inflammation and tissue repair [78]. Plasma cells and B cells, which are also more abundant in the ONH, support local immune responses through antibody production during inflammation or infection [79] (Figure 3).

In short, while many immune and glial cells in the ONH share functional similarities with those in the retina, key differences—such as the unique features of ONH astrocytes, the reactive nature of ONH microglia, and the higher density of infiltrating immune cells—reflect the specialized demands of this structurally vulnerable region.

### 2.3 Immune Cell Composition and Function in Optic Nerve

According to quantitative studies, the optic nerve contains approximately 0.87 to 1.2 million ganglion cell axons and comprises two main regions: the unmyelinated optic nerve (UON) and the myelinated optic nerve (MON) [80] (Figures 1 and 2). This number correlates with the overall density of immune cells present during pathological conditions [81]. Within the optic nerve, the immune cell composition further diversifies, encompassing oligodendrocytes, astrocytes, microglia, macrophages, and infiltrating T and B cells [82] (Figures 1 and 3). The immune response in the optic nerve plays a crucial role in axonal regeneration and remyelination, particularly through its functions in debris clearance, cytokine and chemokine secretion, and the modulation of inflammation following injury [83, 84].

Oligodendrocytes in the optic nerve are pivotal in both myelination and neuroprotection. These cells are responsible for maintaining axonal integrity and providing metabolic support to axons, thereby reducing the energy requirements for action potential propagation [85]. Moreover, oligodendrocytes protect neurons from cytotoxic and excitotoxic insults [86]. They facilitate lactate transport to axons via monocarboxylate transporters (MCTs), promoting axonal function and survival [21]. Furthermore, oligodendrocytes express glutamate‐aspartate transporter (GLAST), indicating a potential role in the glutamate‐glutamine cycle [87] (Figures 2 and 3).

In contrast to retinal microglia, which are more involved in synaptic pruning and maintaining retinal structure during development, optic nerve microglia are primarily tasked with responding to axonal injury [71]. They play a key role in clearing myelin debris at the lesion site following injuries such as optic nerve crush (ONC), though persistent activation can exacerbate damage [71]. These microglia are also essential for supporting the survival of oligodendrocyte precursor cells (OPCs), although their activation can hinder OPC differentiation into mature oligodendrocytes, which are necessary for remyelination [71]. Unlike retinal microglia, optic nerve microglia are less likely to adopt a rod‐like morphology in response to injury [38, 88]. However, similar to retinal microglia, they have the capacity to produce pro‐inflammatory cytokines and reactive oxygen species, thereby contributing to an environment that is less neuroprotective in comparison to the retina [71] (Figure 3). Direct visualization is possible for retinal microglia, but for optic nerve microglia, activation must be inferred in vivo, typically via Translocator Protein‐Positron Emission Tomography imaging, which uses radioligand uptake as a surrogate for microglial activation [89].

Astrocytes located within the optic nerve exhibit distinct characteristics when compared with their counterparts found in the retina. These differences are evident in both their anatomical origins and their morphological features [90]. Optic nerve astrocytes are type II astrocytes, expressing glial fibrillary acidic protein (GFAP) but not connexin‐43 [91]. These cells originate from oligodendrocyte‐type 2 astrocyte (O‐2A) progenitors derived from ventricular zone neuroepithelial cells [47] and typically exhibit a fibrous appearance, being less complex than retinal astrocytes [50, 73] (Figures 1 and 2). These cells play a critical role in supporting myelinated axons and facilitating communication between neurons. Additionally, these cells contribute to the maintenance of ionic balance and the response to injury within the optic nerve environment [51] (Figure 3). In contrast to astrocytes in the ONH, fibrous astrocytes along the optic nerve express AQP4, further supporting their role in fluid regulation and response to injury [69] (Figure 2). Moreover, GLAST, a Müller cell‐specific marker, is also expressed in the optic nerve. However, these cells are neither astrocytes nor oligodendrocytes, suggesting that they may be precursor cells [92] (Figure 2).

Macrophages within the optic nerve can be classified as either resident or infiltrating cells. These cells have been observed to respond to injury and inflammation, especially their phagocytic activity [60, 61]. T cells, as a component of the adaptive immune system, have been identified as a crucial element in the immune response within the optic nerve, particularly in conditions such as optic neuritis and multiple sclerosis. T cells have been shown to exert neuroprotective effects and play a key role in regulating inflammation [60]. B cells, though less prevalent, have been documented in association with inflammatory conditions affecting the optic nerve [76]. A comprehensive review of the role of glial cells in the optic nerve can be found in other reviews [21] (Figure 3).

In summary, the composition and functional roles of immune cells exhibit significant variation across the retina, ONH, and optic nerve, reflecting their distinct contributions within the visual system. Four key features highlight these differences: (1) Regional specialization: retinal immune cells primarily mediate inflammatory responses and pathogen recognition, whereas ONH and optic nerve immune cells focus more on neuroprotection, repair, and maintaining axonal integrity. (2) Cell function heterogeneity: within a given region, specific cell types exhibit unique functions, although they may also share overlapping roles. (3) Cell function convergence: distinct immune cell types in different locations can fulfill the same functional need within a specific area. (4) Glial versus infiltrating cells: these patterns of regional specialization are predominantly seen in major resident glial cell populations. In contrast, infiltrating immune cells typically demonstrate similar functions regardless of location. Collectively, these features emphasize the spatially distinct yet interconnected nature of immune cell functions in preserving visual system integrity.

## 3 Immune Responses in RGC Axonal Injury Due to High IOP

**Figure 4:**
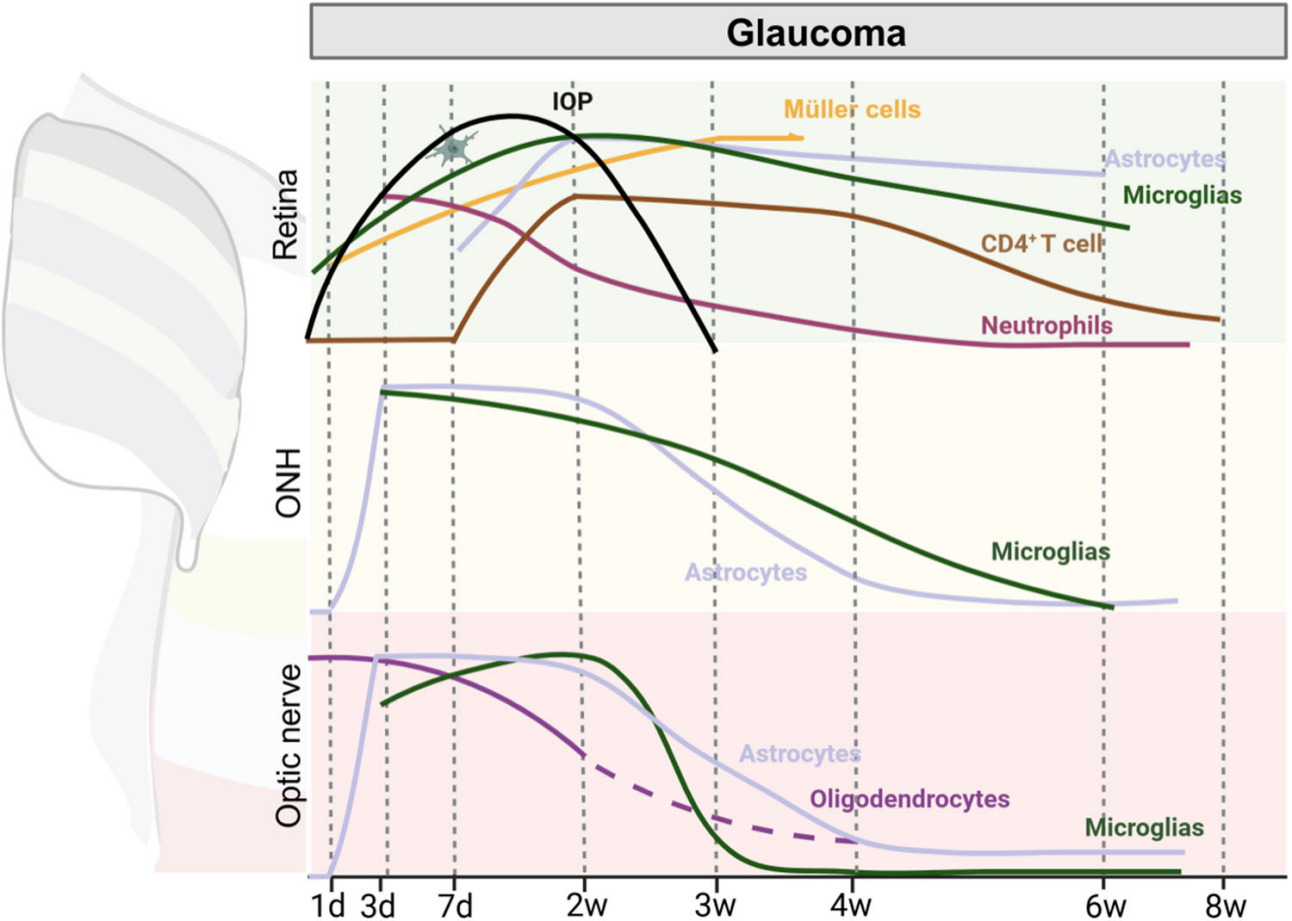
Spatio‐temporal dynamics of immune and glial cells in a microbead‐induced glaucoma model. In this model, intraocular pressure (IOP) increases acutely but transiently post‐microbead injection (postinjection timepoints shown). Retinal microglia activate immediately (Day 1), peaking after the IOP peak (Week 2), and exhibit sustained activation with morphological changes beyond 6 weeks. Microglia in the optic nerve head (ONH) and optic nerve activate similarly early (Day 1), but recovery is fastest in the optic nerve and intermediate in the ONH. Müller cells activate and maintain elevated activity through Week 4. Astrocytes in the ONH and optic nerve activate shortly after microglia activation (preceding retinal astrocyte activation), and recover faster than retinal astrocytes. Neutrophils rapidly infiltrate the retina after microglial activation, but exhibit only transient presence and are not reported in the ONH or optic nerve. In this acute model, retinal immune and glial cells exhibit complex, sustained activation patterns.

**Figure 5:**
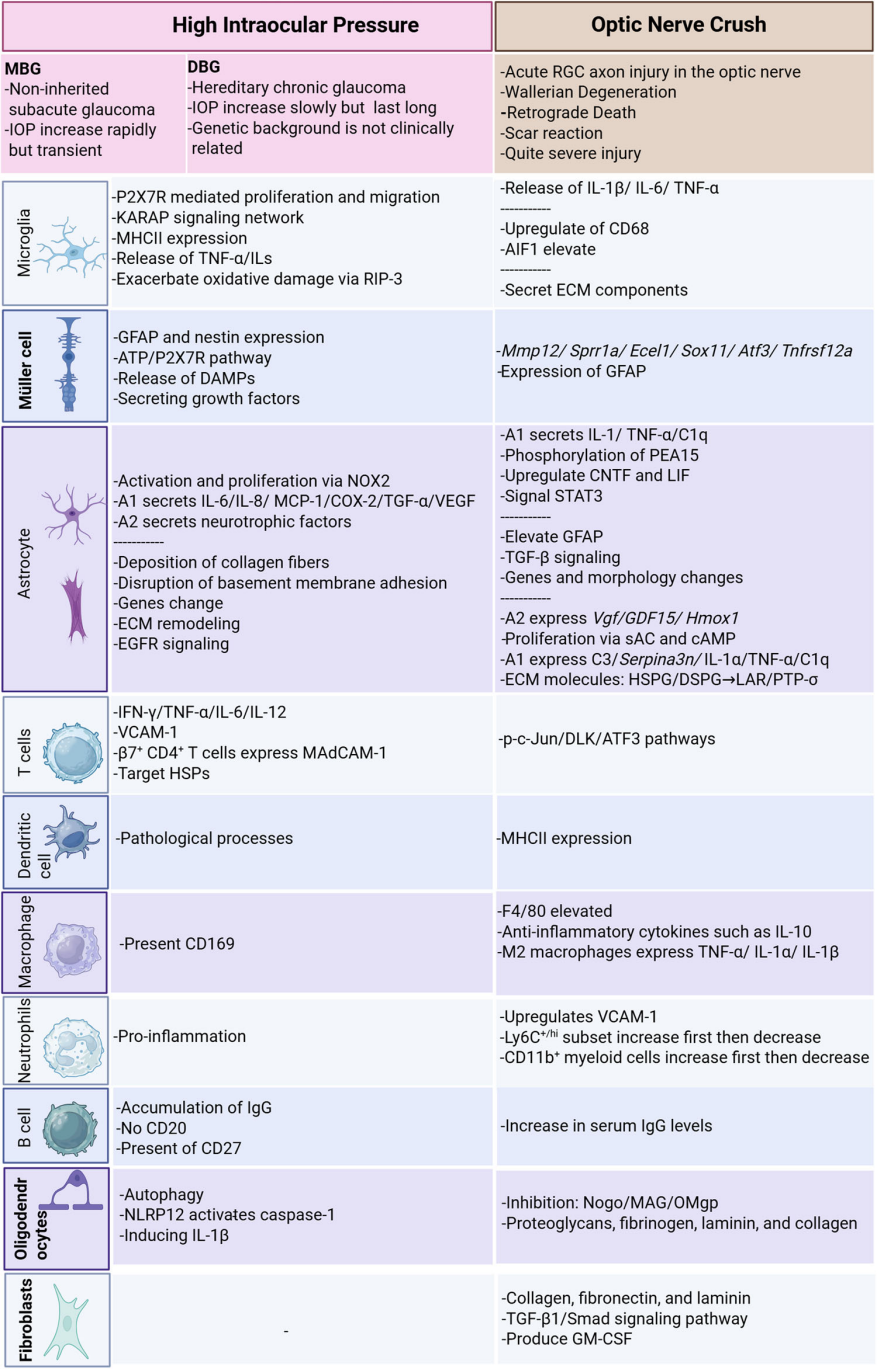
Immune cell function comparison across experimental RGC injury models. Glaucoma models (e.g., MBG, DBG) and optic nerve crush models exhibit key differences in injury location: glaucoma damages somas and axons within the optic nerve head, whereas crush injures distal axons. MBG and DBG themselves differ in the chronicity of intraocular pressure (IOP) elevation. These spatial and temporal contrasts correlate with distinct biological responses. Glaucoma models induce strong retinal immune cell reactivation, while the crush model strongly modulates extracellular matrix (ECM) functions. Furthermore, the specific factors secreted vary significantly between models, highlighting the critical importance of matching disease pathophysiology to identify relevant clinical targets. DBG, DBA/2J mice glaucoma; MBG, microbeads‐induced glaucoma.

**Figure 6:**
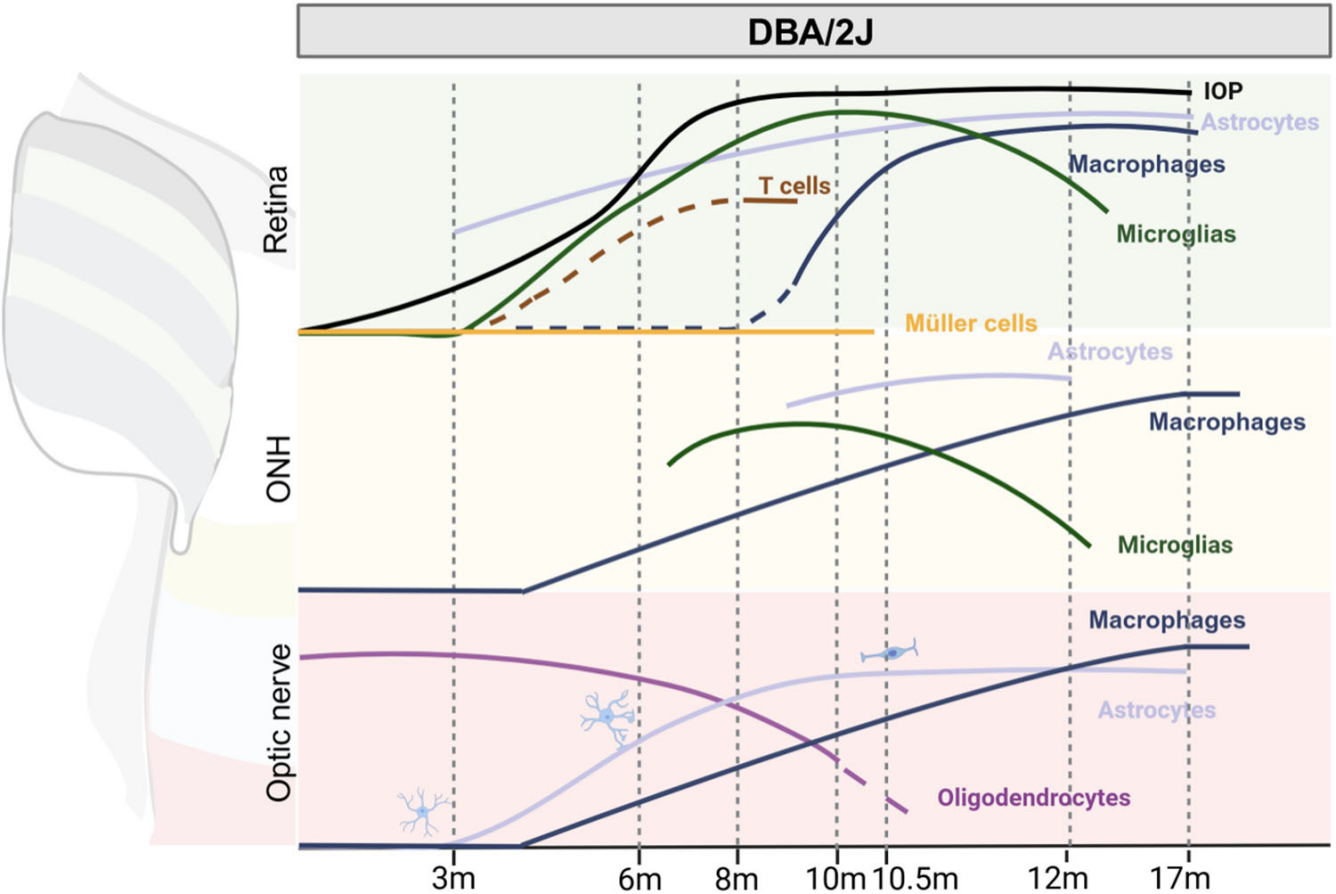
Spatio‐temporal dynamics of immune and glial cells in the DBA/2J mouse model. In this model, intraocular pressure (IOP) increases gradually and consistently. While Müller cells show minimal alterations, astrocytes and microglia activate similarly across the retina, optic nerve head (ONH), and optic nerve. Microglial and astrocyte activation commences soon after the IOP rise at approximately 3 months of age, coinciding with T cell infiltration into the retina. Macrophage infiltration into the retina, however, is delayed relative to that in the ONH and optic nerve, occurring after 8 months of age compared to around 4 months. Twelve months after activation, microglia recover, whereas astrocytes and macrophages remain activated. Notably, astrocytes undergo a morphological shift, transforming from a stellate to a fibrous phenotype.

Glaucoma, a condition marked by elevated IOP, instigates a multifaceted immune response that culminates in RGC axonal injury [93]. The initial phase of elevated IOP is marked by the activation of innate immunity, subsequently giving way to the onset of adaptive immune responses in more prolonged cases [94]. The immune mechanisms throughout glaucoma progression have been extensively reviewed elsewhere [8, 16, 95]. Here, we aim to provide a comprehensive summary of the immune response across various stages and ocular regions according to two predominant glaucoma models. The first model is the microbeads‐induced glaucoma (MBG) model, which exhibits a transient yet pronounced increase in IOP, beginning approximately 1 week after induction [96] (Figures 4 and 5, Table 1). The second model is the DBA/2J mice glaucoma model (DBG), which demonstrates a gradual yet persistent elevation in IOP, commencing at 6–9 months of age [97] (Figures 5 and 6, Table 1). A notable distinction between these two models pertains to the temporal dynamics underlying immune activation, despite their shared functional characteristics.

**Table 1:** Temporal profiles of cell activation in three disease models.

Time	Retina						Optic nerve head				Optic nerve					
	Dendriticcells	T cell	Neutrophils	Microglia	Astrocyte	Müller	Macrophage	Microglia	Astrocyte	Macrophage	Fibroblasts	Oligodendrocytes	T cell	Macrophage	Microglia	Astrocyte
1 day	O	O	O	M, O	O	M, O			M			M, D				M
3 days	O	O	M, O	M, O	O	M, O		M, O	M, O			M, D	O	O	M, O	M, O
5 days	O	O	M, O	M, O	O	M, O		M, O	M, O			M, D	O	O	M, O	M, O
7 days	O	M, O	M, O	M, O	M, O	M, O		M, O	M, O		O	M, D	O	O	M, O	M, O
10 days	O	M, O	M, O	M, O	M, O	M, O		M, O	M, O		O	M, D	O	O	M, O	M, O
14 days	O	M, O	M, O	M, O	M, O	M, O		M, O	M, O		O	M, D		O	M, O	M, O
3 weeks	O	M, O	M, O	M, O	M	M, O		M	M, O		O	M, D		O	M	M, O
4 weeks	O	M	M	M, O	M	M		M	M, O			D		O		M, O
6 weeks	O	M		M, O	M			M	M			D				
8 weeks	O			O								D				
3 months		D		D	D		D					D		D		D
6 months		D		D	D		D	D				D		D		D
8 months		D		D	D		D	D	D	D		D		D		D
10 months				D	D		D	D	D	D		D		D		D
10.5 months				D	D		D	D	D	D		D		D		D
12 months				D	D		D	D	D	D				D		D
17 months					D		D			D				D		D

### 3.1 Microglia

Elevated IOP has been demonstrated to cause increased retinal glutamate, which activates glial cells, thereby disrupting potassium ion transport and reducing membrane protein expression [8]. In the MBG model, retinal microglia are among the first responders, becoming activated within minutes after surgery [27]. This activation is marked by a morphological shift towards a “rod” shape, followed by proliferation and migration that are mediated by purinergic receptor P2X 7 (P2X7R) [98]. The number of microglia increases within 1 day, peaks after 2 weeks (coinciding with significant RGC loss), and remains elevated—though declining—for at least 6 weeks post‐surgery [37, 88, 99]. In a separate study where IOP was sustained for up to 8 weeks, the activation of microglia, characterized by a large cell body and retractable, thick protrusions, but negative for major histocompatibility complex class II (MHCII) expression, was observed 1 week after surgery. The activation of MHCII‐positive microglia occurred 4 weeks following IOP manipulation, and by Week 8 after surgery, the microglia population had increased by 82%, with the majority being MHCII positive, exhibiting reduced protrusions and elongated cells [100]. It is evident that the activation of microglia is closely associated with the duration of IOP elevation. The activation of microglia originates in the retina and subsequently extends to the optic nerve, where activated microglia accumulate at sites of axonal degeneration [37]. In the UON and MON regions of the optic nerve, microglial activation becomes significant later, by Day 3, remains high after 2 weeks, but declines and approaches baseline levels after 6 weeks following IOP manipulation [101], showing a faster decrease and shorter activated duration than retinal microglia. Specifically, in the optic nerve, microglial numbers are significantly elevated for 14 days after surgery, slightly elevated at Day 21 post‐surgery, and return to baseline after 6 weeks following IOP elevation [93, 102], having the shortest activated duration compared with microglial cells in the retina and ONH (Figure 4, Table 1).

In the DBG model, microglial activation patterns differ. In 1‐month‐old mice, retinal microglia show moderate ionized calcium binding adapter molecule 1 (Iba1) expression, with small, localized populations in the inner layers. At 3 months of age, activated retinal microglia become evident, accompanied by an increase in aggregation and Iba1 expression in both the inner and outer retinal layers. This stage is characterized by the absence of elevated IOP or RGC loss. By 4–10 months, microglial numbers undergo a twofold increase [103, 104]. In the ONH, microglial mitochondrial gene expression changes dramatically in conjunction with elevated IOP in the 9‐month‐old mice, though no discernible neurodegeneration is evident. This suggests that altered metabolic processes may underlie these changes. By 12 months, Iba1 levels and microglial numbers significantly decline. It is noteworthy that, in the prolonged presence of elevated IOP, the activation of microglia in the retina and ONH exhibits a high degree of similarity and is less associated with IOP [105] (Figure 6, Table 1).

In both models, the activation of microglia impairs key homeostatic functions, including phagocytosis, inflammation regulation, and cellular sensing. The killer cell activating receptor‐associated protein (KARAP) signaling network is among the first pathways affected [106]. These cells are typically M1 microglia that release pro‐inflammatory cytokines such as tumor necrosis factor‐alpha (TNF‐α) and ILs [107], which increase neurotoxicity by promoting neuronal apoptosis, disrupting the BRB, increasing neuroinflammation, and impairing neuronal signaling. These cytokines can be detected in aqueous humor [108]. These processes also exacerbate oxidative damage and induce apoptosis in Müller cells and RGCs via the receptor‐interacting serine/threonine kinase 3 signaling pathway [109, 110]. Functionally, microglia have been shown to reduce RGC neurite complexity, decrease neural excitability, and release detrimental substances that expedite the degeneration of retinal neurons, thus contributing to the development of blindness in individuals afflicted with glaucoma [111]. Microglia also recruit and activate other immune cells by upregulating chemokines, complement factors, leukocyte adhesion molecules, and MHCII [90]. This immune crosstalk amplifies inflammation and tissue damage, underscoring the central role of microglia in glaucoma pathology (Figure 5). On the other hand, about 7 days after injury, the M2 type gradually increases, promoting the resolution of inflammation and tissue repair. However, if inflammation persists, the M1 type remains dominant for an extended period, thereby inhibiting regeneration [112].

### 3.2 Müller Cells

In response to inflammatory signals from activated microglia, Müller cells are among the earliest responding cells. These cells become activated within 24 h of increased IOP in the MBG model [27, 113], primarily via metabotropic glutamate receptors [16]. The activation of Müller cells is accompanied by an increase in the expression of GFAP, which begins to rise slightly by Day 2 and reaches a sustained plateau by Week 3 post‐surgery [114] (Figure 4, Table 1).

In the DBG model, when labeled with more specific markers such as cellular retinaldehyde‐binding protein and GLAST, Müller cells show no significant changes at 3, 6, or 10 months of age [104]. However, Müller cells undergo a gradual activation process, characterized by a prolonged duration of activation due to their multifaceted functions and prolonged gliotic response [115] (Figure 6, Table 1).

In both models, activated Müller cells undergo a series of morphological changes marked by elevated GFAP and nestin expression. These cells proliferate, increase oxidative stress markers, and release inflammatory mediators [16, 107]. The ATP/P2X7R pathway plays a pivotal role in Müller cell‐induced microglial activation [114], which in turn drives microglia migration and the recruitment of additional immune cells into the retina. Furthermore, Müller cells have been observed to release DAMPs, which have been shown to amplify inflammatory responses and perpetuate cell death cycles [116, 117]. Despite their role in inflammation, Müller cells also provide critical neuroprotective functions. During reactive gliosis, Müller cells support damaged RGCs by secreting growth factors and metabolites, regulating metabolism, and modulating inflammation to promote neuronal survival and functional recovery [115]. In the early stages of high IOP, oxidative stress and apoptosis—mediated by both Müller cells and microglia—are also significant contributors to ONH injury (Figure 5).

### 3.3 Astrocytes

Astrocyte activation in the retina and optic nerve follows a gradual pattern in the MBG model, with significant morphological and functional changes occurring after prolonged exposure to elevated IOP, typically days later [51, 118]. The increase in GFAP‐positive areas is significant by Day 8 post‐surgery, peaks after 2 weeks post‐surgery [107], and remains elevated after 6 weeks following IOP manipulation [93] (Figure 4, Table 1).

In contrast, the DBG model exhibits a more protracted progression of astrocyte activation, characterized by: The onset of GFAP expression in the GCL and NFL occurs at 3–4 months of age, followed by its extension to the IPL by 6 months of age, reaching the INL by 10 months of age, and spreading extensively throughout the retina by 12 months of age [104, 119] (Figure 6, Table 1).

To the aspect of retinal astrocytes' function, a comparison of microglia and astrocytes reveals that the former are activated earlier but only for a shorter duration. The activation of astrocytes is initiated by cytokine signals from microglia [120], with nicotinamide adenine dinucleotide phosphate oxidase 2 (NOX2) playing a key role in their activation and proliferation [107]. Initially, astrocytes adopt a neurotoxic (A1) phenotype, characterized by cell body hypertrophy, extended branches, oxidative metabolism, and limited neuroinflammation relative to microglia [118]. They secrete pro‐inflammatory factors, including IL‐6, IL‐8, and TGF‐α, which contribute to neuronal death, tissue remodeling, and microglial activation [107, 121]. In addition, activated astrocytes surround blood vessels, increasing vascular permeability [107]. Over time, these cells undergo a transition to a neuroprotective (A2) phenotype, characterized by the production of neurotrophic factors that support neuronal survival [121] (Figure 5).

Astrocyte activation in the ONH differs from the retina, as evidenced by the MBG model [22]. Minimal ultrastructural changes are observed at Day 1 post‐surgery; however, substantial changes become apparent by Day 3. Such changes include, but are not limited to, the presence of abnormal extracellular spaces between astrocytes and axons, deposition of collagen fibers, and disruption of basement membrane adhesion, where astrocytes withdraw [122]. At this stage, there is an observed upregulation of genes involved in cell cycle regulation and cell division, as well as inhibitory cell cycle genes [101]. One week after IOP manipulation, significant repositioning of astrocyte processes is observed, accompanied by the extension of abnormal extracellular spaces into the UON. Although GFAP‐positive areas increase, the optic nerve's total area expands, reducing the fraction of GFAP‐positive regions [122]. Minimal changes in gene expression have also been observed [118]. After 2 weeks post‐surgery, astrocyte expression of cell cycle‐related genes diminishes, and oxidative phosphorylation, antioxidant capacity, and protein metabolism are increased, while lipid and steroid metabolism are decreased [101]. After 4 weeks post‐surgery, astrocytes migrate outward from the central region of the lamina cribrosa, and cytoskeletal and ECM remodeling commences, accompanied by partial recovery of transport functions [118, 123]. After 6 weeks post‐surgery, the expression of genes associated with cell cycle regulation and cell division is further reduced, while the expression of genes that inhibit cell cycle is increased [101]. Abnormal extracellular spaces are largely resolved during this period, but new collagen‐rich connective tissue regions form, and ECM damage becomes cumulative and irreversible, altering ONH biomechanics and increasing stress susceptibility [22]. The remodeling of reactive astrocytes and ECM is a continuous process throughout the course of glaucoma. Damage to the ECM is cumulative and irreversible, altering the biomechanical properties of the ONH and leading to increased susceptibility to further stress. Astrocytes also express neuronal precursor markers, similar to retinal Müller cells, reflecting a potential regenerative response [10]. Morphologically, ONH astrocytes exhibit hypertrophy and a general loss of coarse protrusions. They develop long, thin, non‐GFAP‐expressing processes localized to specific regions, contacting only a few RGC axons (Figure 4, Table 1). In the DBG model, complement component 3 (C3) expression in ONH astrocytes increases gradually from 9–10 months to 12 months of age, suggesting a sustained role in glaucoma progression. The activation of C3 has been shown to enhance epidermal growth factor receptor (EGFR) signaling, which in turn regulates the expression of neuroprotective genes in astrocytes [10, 23, 124, 125] (Figures 5 and 6, Table 1).

Astrocyte responses in the optic nerve differ further [126]. In the MBG model, keratin and genes associated with intercellular communication are upregulated by Day 7 following surgery, while oxidative phosphorylation and heat shock transcription factor 4 are upregulated at Week 4 after surgery, accompanied by concurrent downregulation of genes related to ECM, collagen, and intercellular communication [118]. Gene expression changes in the optic nerve are otherwise analogous to those observed in the ONH [101], and astrocytes persist in a state of activation by Week 6 post‐surgery [93] (Figure 4, Table 1). In the DBG model, no optic nerve changes have been observed at 3 months of age [127], but local optic nerve remodeling has been noted at 6 months of age, even in the absence of retinal degeneration. This remodeling encompasses the growth of new branches parallel to nerve fiber bundles and extending into axon bundles, yet it remains confined to specific regions of the astrocytes. At 7–9 months of age, astrocyte processes undergo thickening, a process that is likely attributable to cytoskeletal remodeling and intracellular material deposition. Subsequent to this period, astrocyte processes undergo retraction, resulting in a reduction of spatial coverage and a weakening of connections between nerve fiber bundles. By 10 months and beyond, astrocyte morphology simplifies, with fewer branches and a “rod‐like” shape, further reducing spatial coverage and weakening structural support [126] (Figure 6, Table 1).

### 3.4 Neutrophils and T Cells

In the MBG model, the infiltration of neutrophils into the retina is evident within 3 days post‐surgery, as indicated by a significant increase in Ly6G/Ly6C expression [101]. After 2 weeks following surgery, neutrophil infiltration is significantly reduced, and after 6 weeks, it returns to baseline levels. Similarly, T cell infiltration exhibits a comparable pattern. The expression of CD3, a T cell marker, initially increases but subsequently declines alongside neutrophils, returning to baseline by Week 6 following IOP manipulation [101]. Interactions between retinal microglia and T helper type 1 (Th1) cells engender an inflammatory environment through the release of pro‐inflammatory cytokines such as IFN‐γ, IL‐12, IL‐6, and TNF‐α. Th1 cells also facilitate their own infiltration by upregulating vascular cell adhesion molecule‐1 (VCAM‐1) on retinal endothelial cells. In a separate study, no CD4^+^ T cell infiltration was observed in the retina 7 days after surgery, as IOP had not reached its peak [128]. However, by 2 weeks, when IOP had reached its maximum and RGC loss had reached 17%, CD4^+^ T cells were observed to be evenly distributed in the GCL. Four weeks after surgery, as IOP returned to normal, CD4^+^ T cell numbers began to decline but remained significantly elevated compared to controls, coinciding with further RGC loss (33%). Eight weeks after surgery, despite normal IOP, CD4^+^ T cells remained elevated, and RGC loss reached 35% [96]. The infiltration of β7^+^ CD4^+^ T cells into the GCL occurs 30 days after IOP elevation, inducing the expression of mucosal vascular addressin cell adhesion molecule‐1 in retinal endothelial cells. This observation suggests the existence of a gut‐retina axis, in which endothelial reprogramming in the gut enables β7^+^ CD4^+^ T cells to use integrin β7 for retinal entry, targeting RGCs during circulation and contributing to RGC damage [129]. In sum, neutrophils show an earlier infiltration than T cells; however, T cells have a longer existence window. This discrepancy may be attributed to the presence of various T cell types, which possess a range of functional characteristics. Moreover, neutrophils appear at a rather early phase and decrease quickly (Figures 4 and 5, Table 1).

In the DBG model, the presence of CD4^+^ T cell infiltration and RGC loss are detected at 8 months of age but not at 3 months of age [96]. Transcriptomic changes in RGCs are evident by 9 months, even in the absence of histological changes, indicating early molecular alterations preceding overt disease phenotypes [130]. A notable limitation of the DBG model is the limited duration of T cell function (Figure 6, Table 1).

### 3.5 B Cells

T cells target heat‐shock proteins on the surface of RGCs, indirectly affecting B cell function [96]. This interaction leads to the accumulation of immunoglobulin G (IgG) autoantibodies in the retina, contributing to RGC apoptosis [82]. However, no B cell infiltration has been detected in the retinas of MBG models or 8‐month‐old DBA/2J mice, suggesting that B cell infiltration is not a key factor in the neurodegenerative changes observed in glaucoma [96]. Similarly, no positive immunostaining for CD20, a marker for naïve B cells, has been found in the retinas of glaucoma donors, further supporting the absence of naïve B cells. Interestingly, CD27^+^ cells (indicative of memory B cells or plasma cells) are consistently observed in the retinas of glaucoma patients but not in the retinas of controls [131]. In glaucoma patients, the absence of CD27^+^/CD3^+^ T cells is notable, while the presence of CD27^+^/IgG^+^ plasma cells suggests a potential role for these cells in late‐stage disease progression. These observations, derived from glaucoma donor retinas, suggest that plasma cell activity, rather than B cell infiltration, may contribute to the chronic immune response in glaucoma [131] (Figures 4, 5, 6 and Table 1).

### 3.6 Macrophages

Retinal macrophages are barely detectable in the mouse model of chronic glaucoma at 4 months of age. However, at 10.5 months of age, there is a significant increase in both ramified and round macrophages, with the round type infiltrating from the periphery. This trend is even more pronounced at 17 months of age [132]. A similar pattern is seen in the ONH and optic nerve, where macrophages are absent at 4 months of age but appear as early as 9 months of age [130]. Notably, CD4^+^ T cells are not detected until 8 months of age [133], and macrophage counts continue to increase at 10.5 months of age [134] and up to 17 months of age. In vivo studies in glaucoma patients show that macrophages are confined to the internal limiting membranes and have a highly asymmetric distribution [58]. Early accumulation in the affected area is followed by potential loss as the disease progresses. In addition, infiltration of CD169^+^ macrophages is found in the optic nerves of patients with advanced glaucoma [135] (Figures 5 and 6, Table 1).

### 3.7 Oligodendrocytes

The role of oligodendrocytes in retinal neurodegenerative diseases, such as glaucoma, is a subject of active investigation. While oligodendrocytes may not directly participate in inflammatory responses in glaucoma, they can become targets of inflammatory damage and may undergo demyelination before axonal loss [136]. A study on laser‐induced angle closure demonstrated an 80% decrease in oligodendrocytes in the longitudinal section of the optic nerve 1 week post‐surgery, with a subsequent decline to 55% 2 weeks following surgery. In contrast, a 28% loss of RGCs and axons emerges 3–4 weeks after anterior chamber closure, suggesting that oligodendrocyte degeneration occurs before RGC loss [137] (Figure 4, Table 1). In the optic nerve of 10‐month‐old DBA/2J mice, oligodendrocyte numbers are reduced by approximately 30%. This decline in oligodendrocytes is accompanied by a significant increase in OPCs, whose number rises considerably. This increase in OPCs is not observed at 3 months of age [127] (Figure 6, Table 1).

Chronic inflammation plays a substantial role in the progressive loss of RGCs and optic nerve degeneration in individuals diagnosed with glaucoma [138]. Autophagy is notably activated, particularly within the first 8 weeks after IOP elevation, with autophagy markers shifting from RGC soma and dendrites to RGC soma [76]. Concurrently, NOD‐like receptor family pyrin domain containing 12 (NLRP12) activates caspase‐1, inducing pyroptosis and maturation of IL‐1β, which promotes RGC death and neuroinflammation in glaucoma [139] (Figures 4 and 5, Table 1). In 10‐month‐old DBA/2J mice, the loss of RGCs averages approximately 70% [127, 140]. At 12 months of age, the IOP of DBA/2J mice remains high, and retinal RGC and axonal loss further increases. Germ‐free (GF) DBA/2J mice do not exhibit neurological degenerative changes [96], indicating the critical role of the immune system in glaucoma pathogenesis. This highlights the importance of understanding the immune response in the progression of glaucoma and the potential for targeted therapeutic interventions (Figures 5 and 6, Table 1).

In short, elevated IOP‐induced RGC axonal injury is driven by complex immune mechanisms, progressing from early oxidative stress and complement C3/EGFR signaling to later stages involving autophagy, pyroptosis, and adaptive immune responses. Collectively, these processes contribute to the development and progression of glaucoma. In the mid‐to‐late stages of glaucoma, immune cells, including β7^+^ CD4^+^ T cells, Th1 cells, and B cells, play critical roles. These cells contribute to RGC damage through multiple mechanisms, including the promotion of inflammatory environments, the induction of autoimmune responses, and the direct targeting of neurons.

## 4 Immune Responses in RGC Axonal Injury Due to Trauma

**Figure 7:**
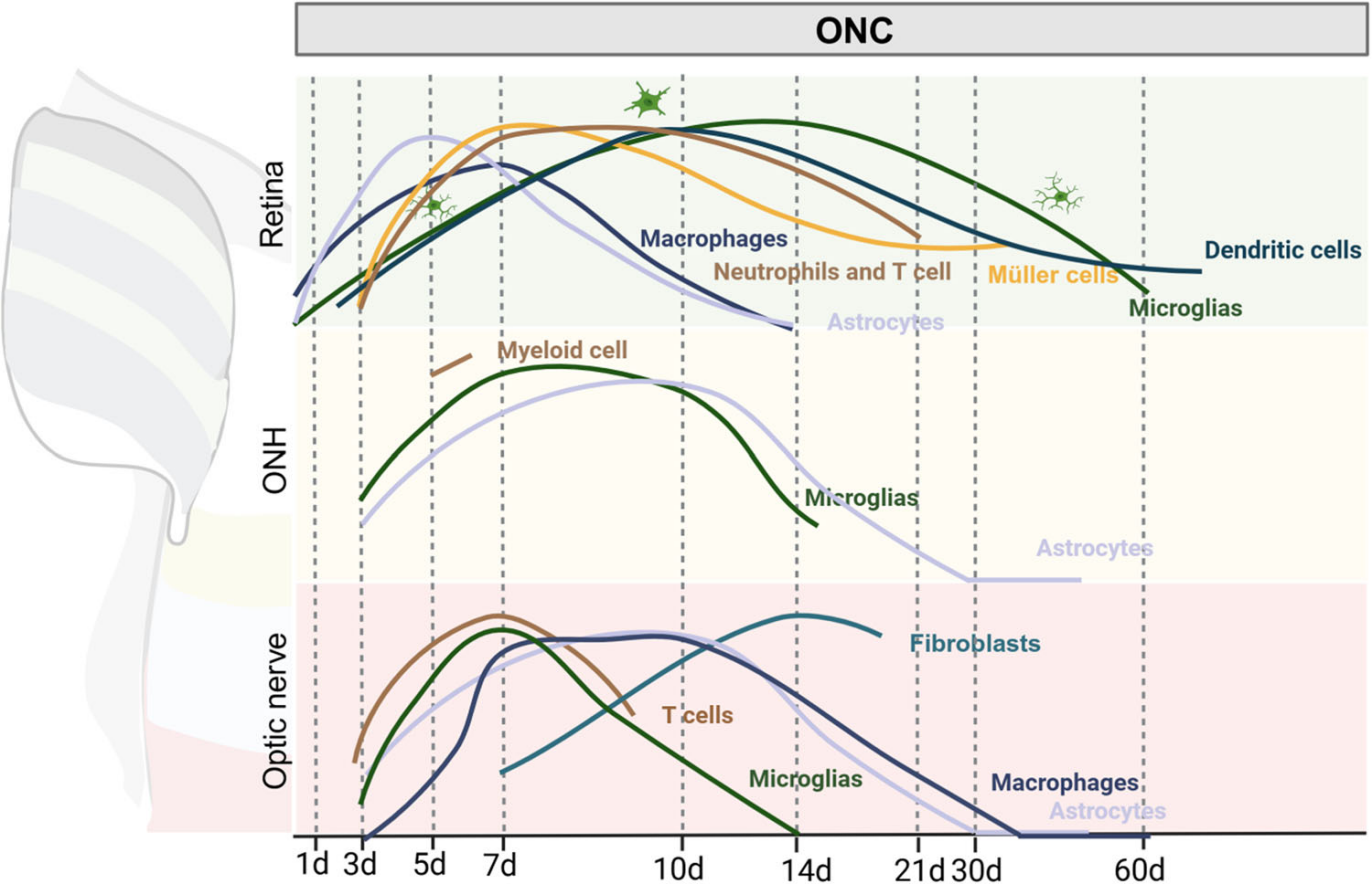
Spatio‐temporal dynamics of immune interactions in the optic nerve crush (ONC) model. Immune responses in the retina begin much sooner than in the optic nerve head (ONH) or optic nerve: within 1 day post‐ONC, macrophages rapidly infiltrate. However, astrocytes and macrophages in the retina recover faster, returning to baseline levels by 14 days after ONC. This swift recovery may stem from the essential function of astrocytes in facilitating scar formation. Conversely, the infiltration of neutrophils and T cells, alongside sustained microglial activation, persists for a more prolonged period within the retina compared to the ONH and optic nerve. This persistent immune activity suggests a compensatory interaction between macrophages and microglia that potentially accelerates retina ganglion cell (RGC) degeneration. Notably, while immune cell populations largely return to baseline in the ONH and optic nerve at 30 days after ONC, microglia and dendritic cells remain activated in the retina. These findings underscore the retina's central importance in orchestrating immune function after injury or challenge and highlight the potential significance of immune communication along the retina‐optic nerve axis for developing therapeutic interventions.

The process of injury and repair in the optic nerve, particularly in cases of traumatic damage or the ONC model, can be categorized into four distinct stages [1]: The acute phase (0–7 days): This phase is characterized by substantial RGC loss and significant morphological changes in the optic nerve [2]; Inflammatory response phase (7–14 days): The inflammatory response intensifies [3]; Regeneration and repair phase (after 14 days): RGCs begin to recover gradually [4]; Long‐term recovery phase (months): This phase extends over several months and is characterized by the gradual restoration of the density and function of RGCs [141]. Concurrently, glial scars and fibrous scars are formed. While the immune response of monocytes around the optic nerve scar has been extensively reviewed [141], this discussion focuses on the responses of other cells as well as glial responses in the retina and ONH (Figures 5 and 7, Table 1).

Retinal cells undergo apoptosis within 48 h after injury, accompanied by morphological changes such as mild edema and vacuolar responses in the optic nerve, as well as glial cell proliferation [142]. Approximately 65% of Brn3a^+^ RGCs are lost within 3 days after ONC, increasing to 70% by Day 14 [143]. Detailed studies have shown that half of the RGCs perish 5.6 days after ONC, while clearance of half of the dead RGCs occurs at 7.2 days. This finding indicates a delay of approximately 3 days between the death of RGCs and the subsequent microglial engulfment [144]. The inflammatory response intensifies gradually during the injury process. This early immune response is critical for initiating repair processes and preventing further neuronal damage [145].

### 4.1 Microglia

After ONC, microglia in the retina are initially distributed uniformly across the RNFL, GCL, IPL, and OPL. These microglia remain in a quiescent state, known as surveying microglia (SMCs), during the first week post‐injury. One week after ONC, SMCs become activated, with their numbers peaking at 14 days before gradually decreasing. Microglial proliferation peaks 2 days after ONC, and the total number of microglia reaches its highest between 5 and 10 days post‐injury [146]. From Day 7 onward, microglia undergo morphological changes, transitioning from branched cells to “rod‐like” or ameboid forms. “Rod‐like” microglia align along RGC axons, while ameboid microglia are primarily involved in phagocytosis of dead RGCs. By 10 days post‐injury, the number of microglia remains elevated; however, their proliferation rate has returned to levels similar to undamaged eyes [146]. After 14 days, microglial morphology begins to normalize, though it remains less complex than in healthy retinas [147]. During the period of rapid RGC death, which extends from 7 days to 2 months post‐injury, microglial activation peaks at around 14–21 days [88]. Approximately 50% of SMCs in the IPL migrate to the GCL in response to RGC death; as RGC loss slows, microglial numbers in the GCL decrease, and some cells return to the IPL. The activated microglia then proceed to engulf the dead RGCs, undergoing a transformation into a state of phagocytic microglia (PMCs) [144]. These PMCs then release pro‐inflammatory cytokines such as IL‐1β, IL‐6, and TNF‐α, which regulate glial scar formation, exacerbate neuroinflammation, and contribute to a neurotoxic environment [21, 148] (Figures 5 and 7, Table 1).

Under normal conditions, microglial markers (e.g., CD68) exhibit low expression in the UON and MON regions [101]. However, significant microglial activation occurs in these areas within 3 days post‐ONC, marked by a substantial upregulation of these markers. Microglial involvement further increases in the ONH between 7 and 14 days post‐ONC, evidenced by a significant rise in microglial numbers and allograft inflammatory factor 1 staining intensity [147]. By 2 weeks post‐injury, microglial marker expression remains elevated in the UON and MON compared to baseline, though levels are reduced from the peak observed at 3 days post‐ONC [147]. Microglia in the optic nerve also contribute to ECM remodeling in the lesion scar after injury [149]. This is reflected in the spatio‐temporal distribution of Iba‐1‐positive microglia, which gradually accumulate from the center towards the periphery of the forming fibrotic scar, particularly by Day 14 post‐injury [150] (Figures 5 and 7, Table 1).

### 4.2 Astrocytes

Increasing evidence indicates that retinal astrocytes interact directly with resident retinal microglia following ONC. The activated retinal microglia secrete pro‐inflammatory cytokines such as IL‐1α, TNF‐α, and the classical complement component 1q (C1q), which induce retinal astrocytes to adopt a neurotoxic A1 phenotype [151]. Among the earliest responders, retinal astrocytes exhibit a significant increase in phosphorylation of phosphoprotein enriched in astrocytes 15 within 6–12 h after ONC [152]. These astrocytes rapidly proliferate and migrate to the injury site, forming a dense network of glial cells [153]. Conversely, by 5 days post‐ONC, reactive astrocytes in the GCL upregulate the expression of ciliary neurotrophic factor (CNTF) and leukemia inhibitory factor (LIF), independent of macrophages. CNTF and LIF activate signal transducer and activator of transcription 3 (STAT3) in RGCs, correlating with a switch of RGCs towards an active regenerative state [154]. Despite this pro‐regenerative role, retinal astrocyte coverage significantly decreases within 1 day after ONC in both injured and contralateral eyes, remaining reduced for 3–7 days. Moreover, the number of astrocytes is significantly reduced by 7 days post‐ONC [155]. However, retinal astrocyte coverage gradually recovers, reaching levels comparable to the infantile retina by 14–21 days post‐injury [156] (Figures 5 and 7, Table 1).

Astrocytes in the UON region, which inherently express elevated levels of GFAP and genes linked to ECM interaction, cell adhesion, and TGF‐β signaling, do not show additional increases in GFAP expression after ONC [101]. By 3 days post‐ONC, astrocytes in both the UON and MON regions display significant morphological alterations: cell bodies and processes enlarge, primary processes diminish, and smaller processes disappear. This coincides with an approximate eightfold reduction in spatial coverage. Morphometric analysis reveals the width of the largest protrusions nearly triples (1.74 ± 0.38 µm), while the convex hull area and overall spatial coverage drastically decrease. Furthermore, these astrocytes upregulate genes related to cell cycle regulation and division. By Day 7, partial reextension of astrocyte processes occurs, accompanied by the swelling of coarse protrusions (width increasing to 2.26 ± 0.44 µm) and a slight increase in the number of primary processes. However, despite these changes, convex hull area and spatial coverage show only marginal improvement and remain significantly below baseline levels [157]. At 2 weeks post‐ONC, gene expression shifts: cell cycle regulation and division genes decline while cell cycle inhibitory genes increase. Morphologically, astrocytes gradually re‐extend their processes, partially restoring their original shape and process length; coarse protrusions' width decreases to 1.77 ± 0.34 µm, and convex hull area and spatial coverage improve, though still not reaching baseline values. Astrocyte morphology is largely restored by 1 month post‐ONC, with protrusion thickness and numbers approaching normal. Nevertheless, protrusion length remains comparatively reduced, resulting in persistently diminished spatial coverage, indicating that full structural recovery has not yet been achieved [157] (Figures 5 and 7, Table 1).

Astrocyte responses in the optic nerve evolve through distinct temporal and functional phases following ONC. By 3 days post‐ONC, a subset of astrocytes, designated as protective A2 astrocytes, emerges. These highly proliferative cells express neurotrophic factors and other genes promoting neuronal survival and regeneration. Their proliferative state is evident in the upregulation of cell cycle marker genes, indicating active progression through the G1, S, and G2/M phases. Soluble adenylyl cyclase regulates this proliferation, with nuclear cyclic adenosine monophosphate promoting it and cytoplasmic cAMP suppressing it. Morphologically, astrocytes exhibit significant changes by this stage, including retraction of primary and higher‐order processes, reduced branching, fewer primary processes per cell body, and increased width of the largest processes. These alterations are prominent but less severe than those in the optic papilla [157]. By Day 7 post‐ONC, A2 astrocytes in the optic nerve maintain a proliferative capacity (albeit potentially reduced) and continue expressing neurotrophic and neuroprotective factors correlated with RGC survival. Concurrently, a distinct population of A1 astrocytes arises, characterized by heightened expression of complement component C3 and pro‐inflammatory genes (e.g., *Serpina3n*), which are implicated in lipid‐mediated neuronal death. While most A1 astrocytes are quiescent (expressing G1 phase markers), a minor subpopulation exhibits proliferative and wound healing signatures. The formation of A1 astrocytes can be inhibited by neutralizing antibodies against IL‐1α, TNF‐α, and C1q [158, 159]. Morphologically, astrocytes begin re‐extending primary processes, although finer branches remain unregenerated. The width of major protrusions decreases slightly, and the number of primary processes per cell body modestly increases [157]. In the subsequent weeks (2 weeks to 1 month post‐ONC), astrocyte morphology continues to recover in the optic nerve. Process length and complexity approach near‐normal levels; however, spatial coverage remains incomplete, indicating suboptimal functional restoration. To summarize the morphological progression: the early phase (3 days) involves significant process retraction, cell body enlargement, and reduced spatial coverage; the intermediate phase (7 days) features partial re‐extension of primary processes without, spatial coverage improves marginally, but small branches recovery; and the late phase (2 weeks to 1 month) sees gradual morphological restoration but persistently incomplete spatial coverage [157].

Astrocytes at the injury site of the optic nerve also synthesize and secrete various ECM molecules. These contribute to a physical barrier inhibiting neural regeneration [160]. Astrocytes are the primary producers of chondroitin sulfate proteoglycans (CSPGs) and other ECM components that form the glial scar. Other cell types, including fibroblasts, pericytes, and meningeal cells, also contribute to ECM components such as proteoglycans, fibrinogen, laminin, and collagen, which collectively form a dense ECM that inhibits axonal regeneration [161]. CSPGs, in particular, have been identified as a pivotal factor in this inhibition, exerting their effects through receptors such as leukocyte antigen‐related receptor protein tyrosine phosphatase and protein tyrosine phosphatase sigma [162] (Figures 5 and 7, Table 1).

### 4.3 Müller Cells

Müller cells in the retina become reactive after ONC, thereby contributing to the inflammatory response by producing cytokines that modulate the local environment and influence the behavior of other glial cells [21, 163]. During this period, Müller cells upregulate various genes functioning in processes such as the inflammatory response, cytokine production, and growth factor release [163]. At 7 days post‐ONC, Müller cells show increased expression of GFAP, with fibers extending across the retinal layers, particularly in the GCL. This elevated GFAP expression persists between 14 and 21 days post‐ONC, though it decreases significantly compared to peak levels at 7 days [147]. Furthermore, immune responses exhibit variability between the dorsal and ventral retinas after ONC, correlating with temporal differences in the recruitment and infiltration of inflammatory cells [164] (Figures 5 and 7, Table 1).

### 4.4 Neutrophils

After ONC, the retinal pigment epithelium (RPE) complex and retina undergo significant immune cell infiltration, with the RPE complex being the initial site of this infiltration. Within 8 h post‐ONC, the RPE complex upregulates VCAM‐1 expression, promoting myeloid cell invasion. By Day 1, there is a significant increase in CD11b^+^ myeloid cells, particularly the pro‐inflammatory Ly6C^+/hi^ subset, which is observed, while the Ly6C^−/lo^ myeloid cells are less prevalent. By Day 3, the total number of CD11b^+^ myeloid cells continues to increase, but the proportion of Ly6C^+/hi^ cells begins to decline as Ly6C^−/lo^ cells gradually increase. By Day 7, the total number of CD11b^+^ myeloid cells begins to decrease, with a significant reduction in Ly6C^+/hi^ cells and a continued rise in Ly6C^−/lo^ cells, reflecting a shift from pro‐inflammatory to anti‐inflammatory and reparative states [165].

In the retina, immune cell infiltration follows the RPE complex. Bone marrow–derived immune cells also infiltrate the retina early on, with scattered bone marrow cells observed rolling and attaching to retinal blood vessels within 3–6 h post‐ONC. By 9 h, there is a dramatic increase in rolling, attachment, and infiltration near the ONH. By 24 h, the retina of the injured eye, as well as the shallow retinal layers of the uninjured eye, is infiltrated by neutrophils (Ly6G/Ly6C^+^) with a higher proportion of Ly6C^+/hi^ pro‐inflammatory cells and a lower proportion of Ly6C^−/lo^ cells [166]. On Day 3 post‐ONC, the proportion of CD11b^+^ myeloid cells continues to increase, while the proportion of Ly6C^+/hi^ cells decreases and the proportion of Ly6C^−/lo^ cells increases. By Day 7, CD11b^+^ myeloid cells remain elevated, while Ly6C^+/hi^ cells are significantly reduced, with Ly6C^−/lo^ cells becoming predominant [101, 165]. By 2 weeks post‐ONC, there is a significant decrease in markers of neutrophils (Ly6G/Ly6C) [101]. Furthermore, live imaging reveals that myeloid cells initially accumulate in the RPE complex, subsequently migrating into the retina and localizing near the GCL.

These findings suggest that the RPE complex serves as an entry route for myeloid cells into the retina and that the immune response transitions from an early pro‐inflammatory state to a later anti‐inflammatory and reparative phase.

It is noteworthy that the accumulation of myeloid cells around the ONH is detected by Day 6 post‐ONC, and these cells originate from retinal microglia rather than circulating macrophages [167] (Figures 5 and 7, Table 1).

### 4.5 Monocytes (Iba1^+^)/Macrophages/DCs

The dynamics of macrophage response are also of critical importance after injury. By 24 h post‐injury, monocytes (Iba1^+^) have infiltrated the retina of the injured eye and the shallow retinal layers of the uninjured (contralateral) eye [166]. Monocyte counts show a transient increase on Day 2 post‐ONC, with macrophage numbers reaching their peak on Day 8. During this peak phase (Day 8), macrophages facilitate axonal regeneration through phagocytosis of cellular debris and the release of growth factors [25]. While the number of macrophages persists at a high level on Day 4, they subsequently decline, with studies reporting an undetectable level by 2 weeks post‐injury [168].

DCs, which arise from monocytes, show a significant increase in the ipsilateral retina starting as early as 2 days after ONC. Their numbers peak at Day 9 post‐ONC, showing a nearly tenfold increase compared to baseline levels. Although the population begins to decline after Day 9 post‐ONC, it remains significantly elevated for at least 60 days. Concurrently, DCs exhibit significantly increased MHCII expression throughout this period [57].

Within 2–4 days following ONC, astrocytes and oligodendrocytes are absent from the lesion site, forming a discrete region devoid of both glial populations in the optic nerve. This astroglia‐ and oligodendrocyte‐free area becomes clearly demarcated by Day 8 post‐injury and persists through Day 18. Although the distal segment of the damaged optic nerve initially shows no significant increase in F4/80‐positive cells relative to baseline, their numbers rise substantially by Day 7, reaching levels approximately four times higher than normal [169]. By Day 12 post‐ONC, nucleated cells, including CD68‐positive blood‐derived macrophages [170], populate the lesion. While sparse immediately after ONC, these macrophages accumulate densely within GFAP‐negative areas between Days 7 and 14, peaking from Days 7 to 10 [150, 171]. During this phase, these macrophages undergo morphological changes characterized by enlargement and robust protrusions, coinciding with active proliferation. Although F4/80‐positive cell counts remain significantly elevated at 3–4 weeks post‐ONC, they begin a gradual decline [169], returning to near‐baseline levels by Day 42. This innate immune response, driven predominantly by macrophages and microglia, is essential for debris clearance and early tissue repair. Key regulatory roles are attributed to anti‐inflammatory cytokines such as IL‐10, which suppresses inflammation, while TNF‐α, IL‐1α, and IL‐1β associate with M2 macrophages [40] that promote tissue repair. Collectively, these dynamics highlight the complex interplay between inflammation and repair mechanisms following ONC (Figures 5 and 7, Table 1).

### 4.6 T Lymphocytes and B Cells

Within 24 h of ONC, T lymphocytes (CD3^+^) infiltrate the retina of the injured eye and the shallow retinal layers of the uninjured contralateral eye [166]. Notably, B lymphocytes (CD19^+^) were absent in these areas at this time point [166]. However, by 2 weeks post‐ONC, T cell marker levels (CD3) decrease significantly [101].

In the optic nerve, T cell infiltration begins at the injury site by Day 3 post‐injury, peaks at Day 7, and remains localized to the lesion area without apparent antigen selectivity [172]. This local infiltration coincides with a later rise in serum IgG levels, suggesting a potential amplification of systemic immunity. Cytotoxic T cells among these infiltrates significantly exacerbate RGC loss via the release of granzymes and perforin [24].

Despite ongoing immune‐mediated damage, surviving RGCs persistently express injury‐response signals, even 56 days after injury. This demonstrates that RGCs retain the capacity to sense axonal severance long after the initial trauma. Together, these observations underscore the protracted interplay between the immune response and neuronal injury following optic nerve damage (Figures 5 and 7, Table 1).

### 4.7 Fibroblasts

After optic nerve injury, fibroblasts play a central role in the process of wound healing and scar formation [173, 174]. In the early stages of injury, fibroblasts are activated, proliferate, migrate, and aggregate at the injury site [173]. Their numbers increase more than 25‐fold by Day 9 and peak around Day 14 post‐injury. By Day 7, the secretion of fibronectin by fibroblasts commences, and by Day 14, a dense fibrous scar is formed, primarily composed of fibroblasts and ECM components such as collagen, fibronectin, and laminin [175]. Fibroblasts secrete collagen, a protein that plays a crucial role in wound closure. This process involves the release of growth factors through mechanical processes that promote the expression of contractile proteins. These proteins serve to anchor the fibroblasts to the matrix, thereby initiating the process of wound contraction [176]. However, an imbalance in fibroblast proliferation and apoptosis can lead to excessive collagen deposition and fibrosis. For instance, in pathological conditions such as keloids, fibroblasts exhibit increased density and proliferation but reduced apoptosis, resulting in excessive collagen matrix deposition and cytokine overproduction. The TGF‐β1/Smad signaling pathway plays a pivotal role in this process, with Smad2/3 promoting fibrosis and Smad7 acting as an inhibitor [177]. Furthermore, fibroblasts contribute to pathological scar formation by persistently residing at the injury site and regulating differentiation and apoptosis through the secretion of cytokines, mechanical stress, and cell‐matrix and cell‐cell interactions. Additionally, activated fibroblasts produce granulocyte‐macrophage colony‐stimulating factor (GM‐CSF), which, in turn, activates macrophages that express Galectin‐3 [174]. This process reaches its peak at 7 days post‐injury and gradually diminishes thereafter. This fibroblast‐macrophage interaction underscores the critical role of fibroblasts not only in wound healing but also in the formation of fibrous and pathological scars following optic nerve injury (Figures 5 and 7, Table 1).

### 4.8 Oligodendrocytes

In the phase following optic nerve injury during which regeneration and repair occur, RGCs begin to recover gradually. Although the process of axonal regeneration in mammals is intricate and less efficient compared to nonmammalian species, some studies have documented axonal sprouting and attempts at regrowth during this period [178]. Long‐term recovery involves synaptic refinement and the functional integration of regenerating axons into existing neural circuits. However, the inhibitory properties of oligodendrocytes present a major challenge to successful axonal regeneration. This inhibition is mediated by specific molecules, including Nogo, myelin‐associated glycoprotein, and oligodendrocyte myelin glycoprotein [179]. These molecules transduce inhibitory signals through a shared receptor complex, thereby creating a biochemical barrier that limits axonal regrowth and functional recovery. Addressing these inhibitory signals is a critical focus in the effort to enhance regeneration and repair in the mammalian optic nerve. Nerve/glial antigen 2 (NG2)‐glia, the precursor cells of oligodendrocytes, also secrete ECM components (e.g., proteoglycans, fibrinogen, laminin, collagen) forming a dense matrix that inhibits axonal regeneration [149] (Figures 5 and 7, Table 1).

### 4.9 Immune Cells in the Superior Colliculus (SC) and Visual Cortex (V1)

Following ONC, significant astrocyte activation and neuronal degeneration occur in the contralateral SC and V1. In the contralateral SC, GFAP‐positive astrocytes show a marked increase starting 3 days post‐ONC and persisting up to 10 weeks. This activation is concomitant with the onset of degenerative neuronal changes [180]. The most significant time points for astrocyte activation in the SC are at 1 and 2 weeks post‐ONC, aligning with peak neuron loss. Protein markers associated with A1 astrocytes, such as C3 and Serping1, show significant increases at 1 week and 3 days post‐ONC, respectively. In contrast, A2 astrocytes within the same region exhibit increased levels of the S100A10 protein marker from 3 days to 8 weeks post‐ONC, while the A2 marker PTX3 shows a notable increase only at 1 week post‐ONC. These findings suggest that astrocyte activation coincides with neuronal degeneration, which becomes most pronounced at 4 weeks and persists until 10 weeks post‐ONC.

In the contralateral V1 region, GFAP‐positive astrocytes also exhibit a significant increase at 2, 4, and 6 weeks after ONC, further indicating a sustained astrocytic response in regions downstream of the injured optic nerve [180]. Together, these findings highlight the temporal and spatial dynamics of astrocyte activation and neuronal degeneration in the SC and V1, suggesting a prolonged response to optic nerve injury that may influence long‐term recovery and repair processes [180].

In summary, during traumatic RGC axonal injury, the early immune responses are predominantly driven by the innate immune system. This prompt immune reaction enables the initiation of recovery processes, including the clearance of damaged tissue and the creation of a favorable microenvironment for cell recovery. In contrast, the late stages of injury involve adaptive immune responses and the regulation of various molecular pathways, which continue to influence RGC survival and axonal regeneration. These subsequent immune responses encompass the participation of T cells, macrophages, and astrocytes, in conjunction with the activation of inhibitory and regenerative signaling pathways. A thorough understanding of these complex and dynamic immune mechanisms is critical for developing effective therapeutic strategies to enhance functional recovery and visual restoration following RGC injury.

## 5 Conclusions and Perspectives

This review systematically delineates the spatiotemporal specificity of immune responses following RGC damage at different sites (retina, ONH, and optic nerve).

### 5.1 Animal Models and Their Relevance to Human Disease

This review examines two established glaucoma paradigms and an optic nerve trauma model to dissect how site‐specific injury to RGCs evolves temporally and recruits distinct immune populations across the retina, ONH, and post‐laminar optic nerve.

For glaucoma, we selected the MBG model and the DBA/2J mouse model. In the MBG model, intracameral microspheres mechanically obstruct the trabecular meshwork, acutely blocking aqueous humor outflow and causing a transient but marked elevation of IOP. Although IOP eventually normalizes, the sustained initial hypertensive phase faithfully recapitulates the cascade seen in human acute angle‐closure glaucoma. The DBA/2J strain, homozygous for loss‐of‐function mutations in *Gpnmb* and *Tyrp1*, develops progressive, lifelong IOP elevation due to iris atrophy and pigment dispersion. This chronic stress culminates in apoptotic RGC death and optic nerve axon loss, closely mirroring untreated primary open‐angle glaucoma in patients. Notably, clinical glaucoma exhibits greater heterogeneity (e.g., normal‐tension variants), necessitating careful translation to patient‐specific contexts. In contrast, the ONC model replicates severe ocular trauma. It induces rapid, reproducible, and severe lesions to the retrobulbar nerve, serving as an extreme yet tractable proxy for traumatic optic neuropathies. While ONC captures immediate immune responses to axonal injury, its mechanical uniformity may not fully reflect the graded damage observed clinically; thus, results should be interpreted within the context of individual injury profiles.

Collectively, these models provide complementary insights into how the anatomical locus and temporal dynamics of RGC injury orchestrate region‐ and time‐specific immune cascades, precisely the dimensions central to this review.

### 5.2 The Temporal Characteristics of Immune Responses in Different RGC Injuries

The immune responses elicited by RGC damage exhibit significant spatiotemporal heterogeneity across diverse anatomical locations and injury types. For example, in glaucoma models, microglia in the retina undergo rapid activation, persisting for an extended duration, while microglia in the optic nerve demonstrate a comparatively delayed activation and more rapid recovery. In cases of traumatic optic nerve injury, the immune response in the retina is more complex and prolonged, suggesting a critical role for the retina in the early stages of injury. Therefore, precise regulation of the inflammatory response in the retina is imperative for the prevention of RGC damage and the preservation of visual function. Precise interventions at different injury types and time points must consider the timing, cell types, and spatial specificity.

### 5.3 The Precise Targets of Immune Responses In Different RGC Injuries

From a clinical translation perspective, the molecular mechanisms and spatiotemporal dynamics of immune cell subsets outlined in this review provide important theoretical foundations for precision therapies. For instance, modulating the activation state of microglia or inhibiting the release of pro‐inflammatory cytokines may decelerate the degenerative changes in RGCs. In addition, enhancing the function of anti‐inflammatory immune cells (e.g., Treg) could engender a more favorable microenvironment for optic nerve repair. Additionally, the comprehensive regulation of the retinal inflammatory response, in conjunction with precise temporal, cellular, and spatial interventions pertaining to diverse injury types, presents a promising avenue for the development of novel diagnostic biomarkers and therapeutic strategies, as shown in Figure 5. Monitoring the activation states of specific immune cells or the cytokines they secrete has the potential to facilitate earlier predictions of glaucoma or optic nerve damage progression, thereby enabling early intervention strategies. The development of targeted therapeutic strategies that focus on specific immune cell subsets has the potential to enhance the efficacy of conventional treatment methods.

Nevertheless, a critical examination underscores the imperative for a thorough consideration of the spatiotemporal characteristics inherent in immune responses while employing these translational targets. The immune responses triggered by RGC damage in different locations vary significantly in terms of cell types, molecular mechanisms, and temporal progression. As such, there is a need for personalized treatment strategies based on the site of injury, type of damage, and stage of disease. For example, in early‐stage glaucoma, suppressing excessive microglial activation in the retina and the release of pro‐inflammatory cytokines like TNF‐α and ILs is crucial. This can also be achieved using specific pathway inhibitors, such as those targeting P2X7R, MHCII, or KARAP. Conversely, following acute optic nerve trauma, therapeutic goals shift towards enhancing the clearance function of immune cells (such as CD68‐positive cells) and inhibiting excessive glial scar formation through antagonism of ECM components. Furthermore, in the chronic injury stage, therapeutic strategies aimed at promoting neuroregeneration and functional recovery, such as injecting neuroprotective factors (e.g., CNTF, LIF) or modifying STAT3/TGF‐β signaling, may necessitate the regulation of immune cell homeostasis.

### 5.4 Precautions From Animal Models to Clinical Applications

This review focuses on three representative RGC injury models: subacute glaucoma, chronic glaucoma, and optic nerve injury. These models effectively distinguish the spatial characteristics of injury: glaucoma models primarily injure RGC axons at the ONH and cell bodies, while the optic nerve injury model predominantly affects distal axons. The two glaucoma models further provide a temporal distinction, representing subacute and chronic injury phases, respectively. Collectively, these models are invaluable for comprehensively characterizing the spatiotemporal changes in immune cells following RGC injury. Despite these advantages, limitations exist for clinical translation. First, most cited studies utilize mouse models. While there is significant genetic homology between mice and humans, and their RGCs are functionally similar, the murine IOP system differs from that of humans. Consequently, while injury outcomes may resemble human conditions, the precise relationship between IOP elevation and injury onset may not fully align. This necessitates more refined criteria when anchoring injury severity or treatment timing to IOP levels. Second, the optic nerve injury model typically induces severe damage. This contrasts with the often milder injuries resulting from optic neuritis or other genetic neuropathies in patients, limiting its direct clinical applicability. Future work should therefore build on this foundation to characterize the immune spatiotemporal response across a spectrum of optic nerve injury severities. Finally, observed differences in gene expression warrant further validation at the protein level to identify key changing factors. Crucially, validation in human samples will ultimately be required to define the landmark protein changes relevant to human pathophysiology.

In summary, this review provides significant insights for clinical treatment and translational medicine by systematically summarizing the spatiotemporal specificity of immune responses following RGC damage. Future research should further explore the specific molecular mechanisms of immune cells in optic nerve pathway injuries and validate the efficacy and safety of these potential therapeutic strategies through preclinical and clinical studies, aiming to provide stronger support for precision therapies related to RGC damage.

## Author Contributions

**Chong Yang:** writing – original draft, data curation, resources, software, methodology, visualization, investigation. **Xiaoyu Wang:** writing – original draft, software, visualization, resources. **Xihang Ye:** writing – original draft, visualization, investigation. **Ye Shen:** funding acquisition, writing – review and editing, supervision, validation. **Jianping Tong:** writing – review and editing, validation, visualization. **Xuhong Zhang:** funding acquisition, supervision, writing – review and editing, formal analysis, validation. **Yudong Zhou:** writing – review and editing, funding acquisition, supervision, project administration, conceptualization.

## Conflicts of Interest

The authors declare no conflicts of interest.
